# Response of Grain Legumes to Phosphorus Application in the Guinea Savanna Agro-Ecological Zones of Ghana

**DOI:** 10.2134/agronj2017.11.0667

**Published:** 2018-04-05

**Authors:** S. Adjei-Nsiah, B.U. Alabi, J.K. Ahiakpa, F. Kanampiu

**Affiliations:** 1 International Institute of Tropical Agriculture, Tamale, Ghana; 2 Research Desk Consulting Ltd., Kwabenya-Accra, Ghana; 3 International Institute of Tropical Agriculture, Nairobi, Kenya

## Abstract

Grain legumes (cowpea, peanut, and soybean) play important roles in household food and income security in smallholder farming systems in the Guinea Savanna agro-ecological zones of Ghana. However, yields are low, rarely exceeding 600 kg ha^–1^, prompting the need to evaluate responses of grain legumes to P fertilizer applications for two seasons. Conducting P studies is critical to help farmers adopt economic-based recommendations. Treatments evaluated in 2015 for the three crops were (i) farmers’ practice (no input and planted by farmer); (ii) control (no input and planted by researcher), and (iii) triple super phosphate (TSP) fertilizer. However, for soybean, an additional two treatments (inoculant only and inoculant plus TSP fertilizer) were included. In 2016, the treatments were the same, except on-farm demonstrations were not conducted on cowpea. The demonstrations were laid out in a Randomized Complete Block Design with each demonstration rep-resenting a replicate within a region. On average, P-fertilizer application increased yields by 296; 527, and 390 kg ha^–1^ for cowpea, peanut, and soybean grains, respectively. On average over the two seasons, P-fertilizer increased yield by 9.85; 13.00, and 17.56 per kg ha^–1^ kg^–1^ P applied for cowpea, soybean, and peanut, respectively, and these applications were cost effective. Peanut showed little response to P in the Upper East Region compared with a greater response in the Northern and Upper West Regions, suggesting that benefits from P-fertilizer for peanut may be location-specific. On average, rhizobium inoculation increased grain yield by 157 kg ha^–1^ across the three regions and significantly positive effects of inoculation were observed in both seasons. Our results show that substantial increases in grain legume yield may be achieved by applying P fertilizers, but farmers cannot afford them because of their relatively high cost. Planting adapted and improved varieties and using rhizobium inoculants may provide the most economically viable and low risk options for increasing yields of grain legumes in the savanna agro-ecological zones of Ghana

## Core Ideas

- Rhizobium inoculation is effective in increasing soybean yields with little financial cost.
- Grain legumes respond significantly to P-fertilizer application in the savanna agro-ecological zone of Ghana.
- Promiscuous soybean responds well to inoculation in the savanna agro-ecological zone of Ghana.
- A significant number of smallholder farmers could benefit from application of rhizobium inoculants to soybean, both agronomically and economically.

The world’s population is projected to increase from the current 7.3 billion to 9.7 billion people by 2050; most of this increase is expected to occur in the developing world (United Nations, 2015) and this suggests the need to increase food production (FAO, 2014a). In areas of high population densities, there is limited potential for expansion of agricultural land, making sustainable intensification crucial (Pretty et al., 2011; Cook et al., 2015; Vanlauwe et al., 2014). Integration of grain legumes in farming systems offers a potential pathway for sustainable intensification, as they are vital in contributing to soil fertility improvement owing to their ability to fix atmospheric nitrogen, making them an excellent component within the cereal-based farming systems in the Guinea savannas of West Africa.

The contribution of grain legumes to soil fertility in the West Africa Savannas has extensively been reported by several researchers (Sanginga et al., 2001; Yusuf et al., 2006; Adjei-Nsiah et al., 2008). In the past, they were cultivated on subsistence basis as food crops. However, over the past decade grain legume cultivation has assumed commercial importance because of their demand by the agro-processing industry and for human consumption. Grain legumes have excellent nutritional values in terms of protein, amino acids, and micronutrients (Phillips et al., 2003; Gibson and Ferguson, 2008; Belane and Dakora, 2011). The dry haulms after harvest and the husk after threshing the pods are an excellent source of livestock feed for ruminants, particularly in the northern Guinea Savanna zone.

Cowpea (*Vigna unguiculata* L. Walp), peanut (*Arachis hypogea* L.), and soybean (*Glycine max* L.) are the three most important grain legumes in West Africa, where they form major components of the predominantly cereal-based farming systems. The Food and Agriculture Organization (FAO) estimates for the 2014 world total area under cultivation of these three legumes was 12.6 million, 26.5 million, and 118 million ha for cowpea, peanut, and soybean, respectively. The world total production of these crops for the same period was about 5.6 million, 43.9 million, and 300 million tons annually for cowpea, peanut, and soybean, respectively. While ~85 and ~23% of the total world production of cowpea and peanut is estimated at 4.5 and 6.5 million tons, respectively, for cowpea and peanut occurs in West Africa, only about 0.26% of the world’s total soybean production occurs in West Africa (FAO, 2014b). These crops are grown mainly for food and cash income but in the dry savannas of Africa, the haulms and husk after harvest are excellent sources of quality feed for livestock and are traded by farmers for cash or exchanged for manure, especially during the dry season.

Legumes play an important role in household food and income security in smallholder farming systems in the three northern regions of Ghana, where about 95% of the total national production of grain legumes occurs (MoFA, 2012). Furthermore, in smallholder farming systems in northern Ghana, grain legumes improve soil fertility characteristics by fixing and supplying nitrogen through return of haulms to the soil after harvest if they are not fed to livestock.

Despite their importance, yields of grain legumes are far below their potential. According to FAO (2014a), the average yield of cowpea in West Africa is estimated at 0.42 t ha^–1^, which is far below the yield of 2 t ha^–1^ achieved on research stations in parts of West Africa (Karikari et al., 2015). Peanut and soybean yields in West Africa are estimated at 1.1 and 0.95 t ha^–1^, respectively. Use of low yielding varieties, low soil P, and high cost of inputs (seeds, fertilizers, and inoculants), have been suggested as the causes for the low yields of grain legumes (Kamara et al., 2007; Kolawole, 2012). Legumes are often considered as secondary crops to cereal crops, and thus commonly promoted as crops that require no fertilizer application. Soils in most parts of the savanna agro-ecological zones of Ghana where grain legumes are cultivated have low fertility. This situation is aggravated by nutrient depletion through continuous cultivation with inadequate replenishment.

Grain legumes can access atmospheric N through symbiosis with soil inhabiting bacteria collectively known as rhizobia and, therefore, require minimal N fertilizer inputs. However, this process can be limited by low availability of other nutrients, particularly P. Several studies have shown that legume yields can be improved using high yielding crop varieties (Buruchara et al., 2011), P-based fertilizers (Kamanga et al., 2010; Kamara et al., 2007), rhizobia inoculants (Sanginga et al., 2000; Osunde et al., 2003), and/or their combinations (Ronner et al., 2015).

During the past decade, several initiatives including the use of high yielding varieties, phosphate-based fertilizers, and inoculants have been introduced to farmers in the three northern regions of Ghana to improve the productivity of grain legumes (Ahiabor et al., 2014; Aziz et al., 2016). While these initiatives are being promoted by research and extension and other projects, their economic viability has not been assessed under farmers’ field conditions. The objective of the present study was, therefore, to evaluate the response of the three commonly cultivated legumes in north-ern Ghana to P-fertilizer and to assess the economic viability of P-fertilizer use to guide farmers in the regions towards profitable adoption of introduced grain legume technologies.

## MATERIALS AND METHODS

### Study Sites

**Fig. 1:**
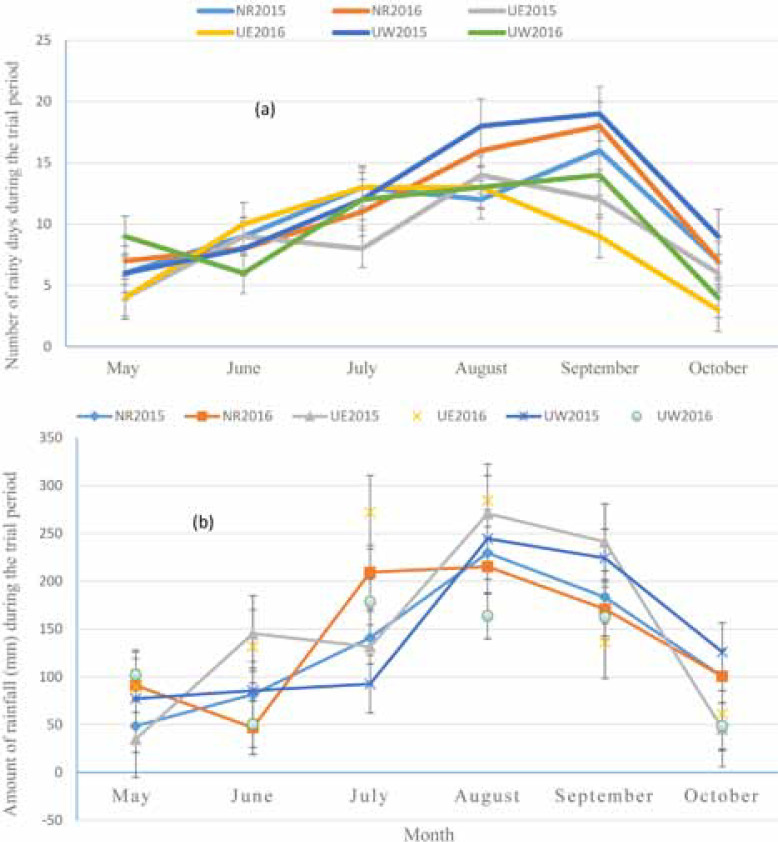
Rainfall distribution in the three study sites during the trial period: (a) number of rainy days and (b) rainfall amounts. The error bars represent standard errors of deviation (SED). NR2015 and NR2016 = number of rainy days or amount of rainfall (mm) in 2015 and 2016 for Northern Region; UE2015 and UE2016 = number of rainy days or amount of rainfall (mm) in 2015 and 2016 for Upper East Region; UW2015 and UW2016 = number of rainy days or amount of rainfall (mm) in 2015 and 2016 for Upper West region.

The study was conducted in three northern regions of Ghana: the Northern and Upper West Regions in the southern Guinea Savanna zone and the Upper East Region in the northern Guinea Savanna zone in the 2015 and 2016 cropping seasons. Rainfall in the study areas is unimodal and occurs between May and October with a dry period between November and April. Northern Region is the wettest of the three regions with a total annual mean rainfall of about 1163 mm while the Upper East is the driest with annual rainfall of 900 mm. The rainfall distribution (number of rainy days and total amounts) for the three sites during the trial period for the 2 yrs is presented in Fig. 1.

The soils in the trial sites (Table 1) are classified as Savanna Ochrosol and Groundwater Laterites in the interim Ghana soil classification system (Adjei-Gyapong and Asiamah, 2002) and as Plinthosols in the World Reference Base for soil resources (WRB, 2015). These soils which are formed under the influence of ground water and lateral water flows from adjacent uplands are poorly drained with iron accumulations that irreversibly harden during repeated drying and wetting to form iron concretions and hard pans. These soils are shallow and, therefore, inhibits root penetration and induce temporal water logging during heavy rains. The shallow depths of these soils and the abundance of iron pans and concretions limit the agricultural potential of these soils. The chemical and physical soil properties of the demonstration plots are presented in Table 1.

**Table 1:** Physicochemical properties of the soils (0–20 cm depth) used for the demonstrations for the three regions

Region	pH	Organic C (%)	Total N (%)	Available P (mg kg^–1^)	Exchangeable cations (cmol kg^–1^)			Texture (%)		
					Ca	Mg	K	Sand	Clay	Silt
Northern	6.4	0.70	0.07	6.87	4.27	1.21	0.18	58	23	19
Upper West	6.2	0.48	0.05	6.50	2.64	1.09	0.14	68	18	14
Upper East	5.8	0.42	0.05	8.67	3.04	1.14	0.13	66	19	15

Major crops cultivated in the three regions include rice, maize, sorghum, millet, yam, peanut, and cowpea. Soybean is emerging as an important cash crop in the three regions.

### On-Farm Demonstrations of Grain Legume Technologies

**Fig. 2:**
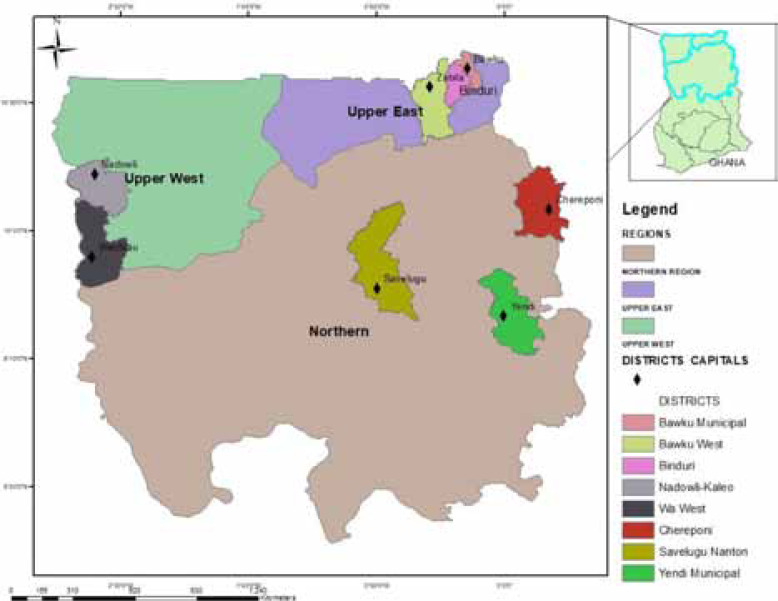
Districts where demonstrations were conducted in 2015 and 2016 in Northern Ghana

As part of the Bill and Melinda Gates Foundation sponsored project, “Putting Nitrogen Fixation to work for smallholder farmers in Africa” (www.N2Africa.org), dissemination campaigns were undertaken both in 2015 and 2016 cropping seasons. The number of households that participated in the dissemination campaigns of grain legumes technologies in the Northern, Upper West, and Upper East Regions in 2015 were 25, 18, and 25 for cowpea, peanut, and soybean, respectively. Eight districts participated in the 2015 dissemination campaign, comprised of three districts from the Northern Region, three districts from the Upper East, and two districts from the Upper West Region (Fig. 2). These districts were selected based on their potential for the cultivation of one or more of the three grain legumes after consultations with local partners. In 2016, dissemination campaigns were conducted on only peanut and soybean and the number of households that participated in the dissemination campaigns in the three regions were 28 and 40 for peanut and soybean, respectively.

All the districts that took part in the 2015 dissemination campaigns (except Chereponi district) participated in the 2016 campaign. A total of seven districts participated; three districts in Upper East and two each in the Northern and Upper West Regions. No dissemination campaign was undertaken on cowpea in 2016.

In each season, between three to five communities managed by one to three extension agents were selected in each district. Within each community, participating farmers were selected by the extension agent based on the farmer’s interest in the cultivation of one of the three grain legumes, and willingness of the farmer to release his/her field for the demonstration and accessibility to the field (for visibility of the field and possibility of other farmers to visit and learn from the demonstration).

Farmers were organized into groups of 15–30 farmers comprising of a lead farmer (trained directly by the project) and 14–29 satellite farmers who also received training from the lead farmer. In 2015, the treatments evaluated were (i) cowpea: farmers’ practice (no fertilization and planted by farmer), control (no fertilization and planted by researcher), and cowpea fertilized with P and planted by researcher; (ii) peanut: farmers’ practice (no fertilization and planted by farmer), control (no fertilization and planted by researcher), and peanut fertilized with P and planted by researcher; and (iii) soybean: farmers’ practice (no fertilization and planted by farmer), control (no fertilization and planted by researcher), soybean seeds treated with inoculant alone and planted by researcher, soybean fertilized with P alone, and planted by researcher and soybean seeds treated with inoculant and fertilized with P and planted by the researcher. In 2016, the treatments were the same except that no demonstration was undertaken on cowpea.

Plot size was 10 × 12 m with planting distances of 60 × 20, 60 × 20, and 60 × 10 cm, respectively, at two seeds per hill for cowpea, peanut, and soybean. The planting periods of the three legume crops were as follows: Peanut, May to July; soybean, July; and cowpea, July to August. The P was applied as TSP (46% P_2_O_5_) at 30 kg ha^–1^ P within the first 2 weeks of planting. The fertilizer was banded in a 3 cm deep trench 10 cm away from the planting line and covered. The rhizobium inoculant (Nodumax) containing 10^8^ cells g^–1^ of *Bradyrhizobium japonicum* (strain USDA 110) was applied to the seeds at the rate of 7 g kg^–1^ of seeds. The demonstrations were planted by the farmers with the assistance from field extension officers. Management of the demonstrations was done by farmers.

### Data Collection and Analyses

All the demonstrations were monitored during the growing seasons. Information on planting, weeding, and harvest dates; conditions of the field; and the cropping history of the field was recorded in a field book. The field book also contained questions on socio-economic characteristics of the households. Farmers filled the field books with the help of the extension officers. At the end of the season, farmers harvested the plots separately and evaluated the different treatments in the demonstrations. The pods were threshed and the grains kept separately until they were weighed and the weight recorded by the extension agents. Grain yields represent air-dried weight (11–14% moisture content). The data was cleaned to only include demonstrations where yields of all treatments were reported. At the establishment of the demonstrations, soils samples were taken from 0–20 cm depth of the soil from every field. On each field, five soil core samples were taken randomly, thoroughly mixed, and composite samples taken. These samples were air-dried and sieved with a 2 mm mesh and analyzed for pH, organic carbon, total N, P, and exchangeable cations.

An economic analysis was performed on the profitability of use of P-fertilizer for each of the grain legumes. Costs of fertilizer was determined using prices of local TSP fertilizer and labor costs for fertilizer application was based on the existing labor rates in the communities. Price for the grain legumes are the average market price for 2017. All the amounts are expressed in US dollars (USD, $) at the average exchange rate Jan.–Nov. 2017 (4.4 GHC = $1.00) (Bank of Ghana, 2017). The economic analysis were performed using Microsoft excel spreadsheet (vers. 2016) and the graph was plotted using SAS-JMP software (SAS Institute Inc., Cary, NC). The effects of region, P and their interactions on yield were determined by performing analysis of variance (ANOVA) using Genstat statistical software (vers. 12; VSN International Limited, Hemel Hempstead, UK).

## RESULTS

### Soil Chemical and Physical Properties

Both the soil chemical and physical properties at the three locations were generally poor (Table 1) as reported earlier by Buri et al. (2009). However, soils in the Northern Region were relatively better compared with the two remaining regions in terms of pH, total nitrogen, organic carbon, exchangeable cations and clay content. The low organic carbon and total nitrogen contents of the soils in the Upper East Region could partly be attributed to low organic matter and high temperatures that accelerate decomposition of organic matter (Agboola and Aiyelari, 2000).

### Grain Legumes Response to Phosphorus Fertilization and/or Inoculation

#### Cowpea

**Fig. 3:**
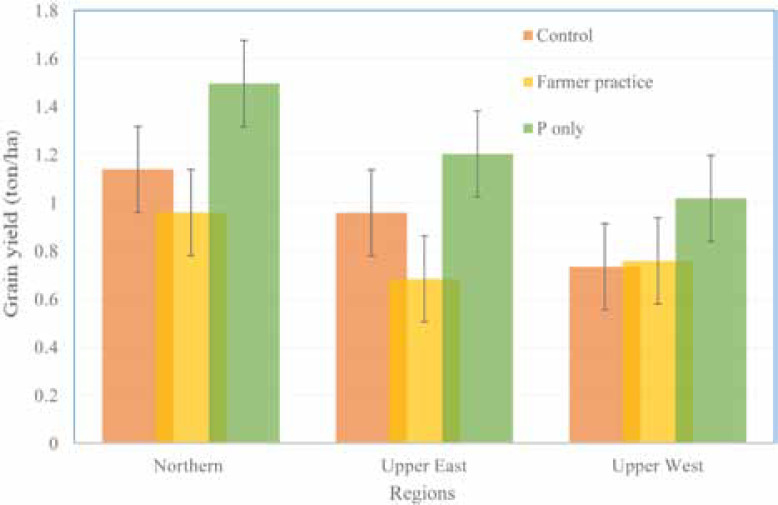
Effect of location (region) and treatment (farmers’ practice [no input and planted by farmer], control [no input and planted by researcher], and triple super phosphate (TSP) (P) (planted by researcher) on cowpea grain yield in on-farm demonstrations in northern Ghana in 2015. Vertical error bars represent SED (standard error of difference between means).

Cowpea grain yield under farmers’ practice was generally low and ranged from 0.68 t ha^–1^ in Upper East Region to 0.96 t ha^–1^ in the Northern Region (Fig. 3). Phosphorus application resulted in the highest grain yields ranging from 1.02 t ha^–1^ in Upper West Region to 1.5 t ha^–1^ in the Northern Region. Grain yields across the three regions ranged from 0.84 t ha^–1^ in the Upper West Region to 1.2 t ha^–1^ in the Northern Region. Response of cowpea to P application in the three regions were 11.93, 9.47, and 8.17 kg ha^–1^ kg^–1^ P application in Northern, Upper West, and Upper East Regions, respectively. Grain yields from the farmers’ practice plots were generally low, particularly in the Upper East and Northern Regions, partly due to low plant population, although we did not take data on plant population.

#### Peanut

Both P application and location had significant effects on peanut pod yields in both 2015 and 2016 (Table 2). In 2015, pod yields from farmers’ practice plots ranged from 0.57 t ha^–1^ in the Upper East Region to 1.93 t ha^–1^ in the Upper West Region while yields from control plots ranged from 0.83 t ha^–1^ in the Upper East Region to 2.04 t ha^–1^ in the Northern Region (Table 2). In 2015, while peanut did not respond to P application in the Upper East Region, the response of peanut to P application were 41.87 and 36 kg ha^–1^ kg^–1^ P applied in the Upper West and Northern Regions, respectively. The pod yield in 2016 followed a similar pattern as that of 2015 with both P application and location significantly influencing pod yield. Across regions, P application resulted in the highest pod yield with the highest yield occurring in the Northern Region and the lowest pod yield occurring in the Upper West Region. Response to P were 38.57; 9.63, and 6.53 kg ha^–1^ kg^–1^ P applied for Northern, Upper West, and Upper East Region, respectively.

**Table 2:** Effect of location (region, R) and treatment (T) (farmers’ practice [no inputs and planted by farmer], control [no input and planted by researcher] and triple super phosphate (TSP) (P) [planted by researcher]) on pod yield of peanut in on-farm demonstrations in northern Ghana in 2015 and 2016.

Region	No. demonstrations	Farmers’ practice	Control	TSP (P)	Mean
Pod yield for 2015 cropping season (tons ha^–1^)					
Northern	10	1.747	2.038	3.118	2.301^a^†
Upper East	3	0.571	0.831	0.831	0.744^b^
Upper West	4	1.925	1.891	3.147	2.321^a^
Mean	17	1.582^a^	1.79^a^	2.721^b^	
LSD (0.05); T = 0.3849, R = 0.5122, and T × R = 0.9163					
Pod yield for 2016 cropping season (tons ha^–1^)					
Northern	10	2.615	3.549	4.706	3.623^b^
Upper East	13	1.144	1.722	1.918	1.594^a^
Upper West	5	0.627	1.155	1.444	1.075^a^
Mean	28	1.577^a^	2.273^ab^	2.829^b^	
LSD (0.05); T = 0.7007, R = 0.8955, and T × R = 1.658					

† Means in the same column and row followed by the same letter are not significantly different (*p* ≤ 0.05).

#### Soybean

Phosphorus application and location significantly influenced soybean grain yield both in 2015 and 2016 (Table 3). In 2015, application of P alone resulted in increases in grain yield of 301; 307, and 577 kg ha^–1^ in Northern, Upper East, and Upper West Regions, respectively, translating into responses of 10.03; 10.23, and 19.33 kg ha^–1^ kg^–1^ P applied. When rhizobium inoculant was applied in combination with P, the response of soybean to P application were 12.46; 9.03, and 10.67 for Northern, Upper East, and Upper West Regions. Rhizobium inoculant application alone resulted in grain yield increases of 275; ^–34^, and 230 kg ha^–1^ for Northern, Upper East, and Upper West Regions, respectively.

**Table 3:** Effect of location (region, R) and treatment (T) (control [no inputs and planted by researcher]), Farmers’ practice (no input and planted by farmer)), I (rhizobium inoculants and planted by researcher), triple super phosphate (TSP) fertilizer (P) (planted by researcher) and P+I (planted by researcher) on grain yield of soybean in on-farm demonstrations in northern Ghana in 2015 and 2016.

Region	No. demonstrations	Farmers practice	Control	I	TSP (P)	+I + P	Mean
Grain yield 2015 cropping season (tons ha^–1^)							
Northern	10	0.853	0.982	1.257	1.283	1.356	1.150^b^
Upper East	6	1.258	1.722	1.688	2.029	1.993	1.738^a^
Upper West	3	0.532	0.465	0.695	1.042	0.785	0.709^c^
Mean	19	0.927^a^†	1.130^ab^	1.301^bc^	1.477^c^	1.463^c^	
LSD (0.05);T = 0.2605, R = 0.2686 and T × R = 0.6338							
Grain yield 2016 cropping season (tons ha^–1^)							
Northern	18	1.546	1.485	1.695	1.857	2.139	1.745^a^
Upper East	14	0.644	0.786	0.976	1.254	1.398	1.011^b^
Upper West	8	0.468	0.360	0.432	0.673	0.739	0.534^c^
Mean	40	0.886^e^	0.877^d^	1.034^c^	1.261^b^	1.425^a^	
LSD (0.05); T = 0.1794, R = 0.1695 and T × R = 0.4011							

† Means in the same column and row followed by the same letter are not significantly different (*p* ≤ 0.05).

In 2016, application of P alone resulted in grain yield increases of 372; 468, and 313 kg ha^–1^ representing 12.4; 15.6, and 10.43 kg ha^–1^ kg^–1^ P applied for the Northern, Upper East, and Upper West Regions, respectively. When Rhizobium inoculant was applied in combination with P-fertilizer, the additive effects resulted in 654;, 612, and 379 kg ha^–1^ increase in grain yield of soybean for the Northern, Upper East, and Upper West Regions, respectively. The resultant responses of soybean to P plus rhizobium inoculant application were 21.8, 20.4, and 12.6 kg ha^–1^ kg^–1^ P applied. In 2016, application of Rhizobium inoculant alone resulted in marginal increase in grain yield, ranging from 72 kg ha^–1^ in Upper West Region to 201 kg ha^–1^ in the Northern Region.

### Grain Legume Response to Phosphorus Application and Benefit/Cost Ratios of Phosphorus Application to Grain Legumes in the Three Northern Regions of Ghana

The current input price of $2.50 kg^–1^ P (and labor for application of $0.95) and cowpea grain price of $0.64 kg^–1^ suggest that, the mean response of 9.85 kg ha^–1^ kg^–1^ P is higher than the 5.39 kg ha^–1^ kg^–1^ P needed to break even. Consequently, the response of 11.9 kg ha^–1^ kg^–1^ P observed in the Northern Region would equate to a benefit cost ratio of 2.21 while the responses of 8.17 kg ha^–1^ kg^–1^ P and 9.47 kg ha^–1^ kg^–1^ P applied obtained in the Upper East and Upper West Region will equate to benefit cost ratios of 1.52 and 1.76, respectively (Table 4). Likewise, for soybean, the input price of $2.50 kg^–1^ P added to the labor cost of $0.95 for applying the fertilizer and the grain price of $0.34 kg^–1^ both suggest that the average response of 13 kg ha^–1^ kg^–1^ P is higher than the 10.15 kg ha^–1^ kg^–1^ P needed to break even. This gives a mean benefit cost ratio of 1.28 (Table 4). For peanut, the average response of 17.56 kg ha^–1^ kg^–1^ P is higher than the 10.15 kg ha^–1^ kg^–1^ P needed to break even giving an average benefit cost ratio of 1.73.

**Table 4:** Grain legume response to P application and benefit cost ratios of P application to grain legumes in the three northern Regions of Ghana

Region	Response to P (kg ha^–1^ kg^–1^ P)			Revenue (USD, $) from kg of P applied†	Cost of kg P applied (USD, $)‡	Cost/benefit ratio
	2015	2016	Mean			
Cowpea						
Northern	11.93	–	11.93	7.64	3.45	2.21
Upper East	8.17	–	8.17	5.23	3.45	1.52
Upper West	9.47	–	9.47	6.06	3.45	1.76
Peanut						
Northern	36.00	38.57	23.67	8.05	3.45	2.33
Upper East	0	6.53	3.27	1.11	3.45	0.32
Upper West	41.87	9.63	25.75	8.76	3.45	2.54
Soybean						
Northern	10.03	12.4	11.22	3.81	3.45	1.11
Upper East	10.23	15.6	12.92	4.39	3.45	1.27
Upper West	19.33	10.43	14.88	5.06	3.45	1.47

† Prices of Cowpea grains, peanut pods and soybean grains are estimated at $0.64, $0.34, and $0.34 kg^–1^, respectively.

‡ Includes cost of labor for fertilizer application of $0.95 kg^–1^ P.

## DISCUSSION

### Grain Legumes Response to Phosphorus Fertilization and/or Inoculation

The study suggests that P application may contribute to increased yield in grain legumes on farms. Yields were generally higher in the Northern Region over the 2-yr period due partly to better soils (Table 1) and better rainfall distribution (Fig. 1). The response to P application, although varying from region to region, shows an average positive effect of 296; 527, and 390 kg ha^–1^ for cowpea grains, peanut pods, and soybean grains, respectively. These values are, however, lower than what has been reported in recent literature (Ronner et al., 2015; Karikari et al., 2015; Kyei-Boahen et al., 2017). On average, yield response to P were 9.85; 13.00, and 17.56 kg ha^–1^ kg^–1^ P applied for cowpea, soybean, and peanut, respectively, over the 2-yr period. The observation that there was little response of peanut to P in the Upper East Region (3.27 kg ha^–1^ kg P) while the response was as high as 23.67 and 25.75 kg ha^–1^ kg^–1^ P applied over the 2 seasons in the Northern and Upper West Regions, respectively, suggests that benefits from P-fertilizer for peanut may be location-specific. The low response of peanut to P in the Upper East Region may partly be due to late start and early cessation of rains in the region (Fig. 1), which might have resulted in poor pod development and hence low yields. Grain legume response to P fertilization has been reported by several authors (Kamara et al., 2007; Mahamood et al., 2009; Kamanga et al., 2010; Kamara et al., 2007; Karikari et al., 2015). The increased yield with P fertilization is attributed to several factors, including increased uptake of micronutrients, rapid plant growth, increased nitrogen fixation, enhanced flowering, and podding as P is known to play important roles in many processes in legumes such as energy transfer, nodulation, atmospheric nitrogen fixation, flower initiation, fruit development, and seed formation (Beegle and Durst, 2002; Krasilnikoff et al., 2003; Ndakidemi and Dakora, 2007; Nyoki et al., 2013).

The yield response of soybean to rhizobium inoculation, although variable, shows an average positive effect of 157 kg ha^–1^ during the two seasons. While this value is lower than what was reported by Ronner et al. (2015) in northern Nigeria, it is consistent with what has been reported in sub-Saharan Africa recently by van Heerwaarden et al. (2017). The soybean variety used in this study was the promiscuous type, yet it responded to inoculation. This confirms an earlier report by Ronner et al. (2015) that promiscuous soybean varieties respond to inoculation in the savanna agro-ecological zones of West Africa. This is contrary to earlier report that promiscuous soybeans do not respond to rhizobium inoculation (Okungu and Sanginga, 2003). The low cost of rhizobium inoculant which is currently sold at $12.50 ha^–1^ in Ghana means that even with grain prices as low as the current $0.34 kg^–1^, the observed mean response would translate into a net benefit of $40.88 ha^–1^ and a benefit cost ratio of 3.3. Thus, a great majority of farmers could benefit from application of rhizobium inoculants to soybean both agronomically and economically, as they are cheaper. Thus, rhizobium inoculation is effective in increasing soybean yields with little financial cost provided farmers have access to good quality inoculants. Unfortunately, this input is not widely used in Sub-Saharan Africa, due to low level of awareness and inaccessibility to good quality inoculants in rural areas.

## CONCLUSION

This study has shown a widespread response of grain legumes to P fertilizer application and soybean to rhizobium inoculation in the Guinea Savanna agro-ecological zones of Ghana. While these inputs appear to result in high benefit cost ratio, they are not widely used by farmers due to low level of awareness, high cost, and their unavailability in rural areas. This suggests the need to facilitate public-private partnerships to commercialize these inputs and make them readily available in rural farming communities in the Guinea Savanna agro-ecological zones of Ghana in a sustainable way. The low response of peanut to P application in the Upper East Region over the two seasons suggests that response of peanut to P may be location-specific and, therefore, may not be profitable for farmers to invest in this input in all locations. Moreover, while P application may contribute to the productivity of grain legumes with a high benefit cost ratio, its high cost may preclude some farmers from using it. Therefore, planting more adaptable improved grain legume varieties and using rhizobium inoculants may provide an economically viable and low-risk option for increasing grain legumes yields in the savanna agro-ecological zones of Ghana.

## Abbreviations

TSP,: triple super phosphate
